# Protein kinase A signaling regulates immune evasion by shaving and concealing fungal β-1,3-glucan

**DOI:** 10.1073/pnas.2423864122

**Published:** 2025-06-09

**Authors:** Arnab Pradhan, Olga A. Nev, Ian Leaves, Oleg A. Nev, Qinxi Ma, Gillian Milne, Grace Patterson, Mihai G. Netea, Lars P. Erwig, Rhys A. Farrer, Gordon D. Brown, Hugo A. van den Berg, Neil A. R. Gow, Alistair J. P. Brown

**Affiliations:** ^a^Medical Research Council Centre for Medical Mycology at the University of Exeter, Department of Biosciences, Faculty of Health and Life Sciences, Exeter EX4 4QD, United Kingdom; ^b^Software Consultant, Belgrade, Serbia; ^c^Microscopy and Histology Facility, Institute of Medical Sciences, University of Aberdeen, Aberdeen AB25 2ZD, United Kingdom; ^d^Bioimaging Centre, Department of Biosciences, University of Exeter, Exeter EX4 4QD, United Kingdom; ^e^Department of Internal Medicine and Radboud Center for Infectious Diseases, Radboud University Medical Center, Nijmegen 6525 GA, The Netherlands; ^f^Department for Immunology and Metabolism, Life and Medical Sciences Institute, University of Bonn, Bonn 53115, Germany; ^g^Cancer Research UK, London E20 1JQ, United Kingdom; ^h^Mathematics Institute, University of Warwick, Coventry CV4 7AL, United Kingdom

**Keywords:** pathogen-associated molecular patterns, β-glucan masking dynamics, *Candida albicans*, mathematical modeling

## Abstract

Fungal pathogens infect billions and kill millions of people each year. Many of these pathogens have evolved strategies to evade our antifungal immune defenses. *Candida albicans,* for example, masks the proinflammatory pathogen-associated molecular pattern (PAMP) β-1,3-glucan, in response to specific host signals such as lactate. In *C. albicans*, most β-1,3-glucan lies in the inner cell wall shielded, by the outer mannan layer, from recognition by certain immune cells such as macrophages. β-1,3-glucan that becomes exposed at the cell surface can be shaved off by secreted enzymes. By integrating mathematical modeling with experimentation, we show that the dynamics of this shaving, together with the dynamics of β-1,3-glucan exposure during growth, can account for a range of β-1,3-glucan masking phenotypes. The mathematical model accurately simulates the dynamics of β-1,3-glucan exposure during growth and predicts levels of β-1,3-glucan shaving under a variety of conditions, revealing how subtle differences in growth contribute to observed variabilities in lactate-induced β-1,3-glucan masking. For example, clinical isolates previously thought to display minimal lactate-induced masking are shown to mask robustly. Using a range of *C. albicans* mutants, we confirm the importance of Gpr1/Gpa2-protein kinase A signaling for lactate-induced β-1,3-glucan shaving and define the contributions of the Xog1 and Eng1 glucanases to this shaving. Furthermore, examination of a shielding-defective *C. albicans mnn2*x6 mutant confirms that both β-1,3-glucan shaving and shielding contribute to the dynamism of β-1,3-glucan masking at the fungal cell surface. Dynamism in PAMP masking is likely to be relevant to other fungal pathogens of humans.

## Significance

Pathogenic fungi impose a heavy burden upon human health. Many, particularly opportunistic fungal pathogens such as *Candida albicans*, have evolved strategies to evade our immune defenses. These include masking of pathogen-associated molecular patterns (PAMPs) whose recognition triggers antifungal immunity. *C. albicans* masks the proinflammatory PAMP, β-1,3-glucan from recognition by macrophages, thereby turning this fungus into a moving target for immune cells. Combining experimentation with mathematical modeling to explore the dynamics of this phenotype, we show that β-1,3-glucan exposure during growth, combined with β-1,3-glucan shaving, underlie this PAMP masking. We define how different host-related signals impact β-1,3-glucan exposure by influencing growth and shaving, providing insights that are relevant to immune evasion by other fungal pathogens.

Fungal infections have been estimated to kill more than two million people each year (1). The importance of antifungal immunity in protecting against lethal fungal infections is highlighted by the particular susceptibility of immunocompromised individuals to such infections (2). Innate immune cells recognize fungal pathogens via pathogen-associated molecular patterns (PAMPs), some of which are located at the fungal cell surface (3–5). However, fungal pathogens deploy a variety of strategies to evade or avoid immune recognition and clearance (6–8). These strategies, which include the masking of proinflammatory PAMPs (9–12), permit some fungi to modulate the nature of the immune response.

*Candida albicans* is a commensal fungus that colonizes skin and/or mucosae (13–15). During its coevolution with the host, this opportunistic fungal pathogen has developed the capacity to exploit host signals such as lactate, hypoxia, ambient pH, and iron limitation to induce masking of the major proinflammatory PAMP, β-1,3-glucan at its cell surface (9, 10, 16, 17). β-1,3-glucan masking reduces fungal recognition and killing by innate immune cells such as neutrophils and macrophages (9, 10, 16, 17) and compromises host clearance of *C. albicans* infections (18).

β-1,3-glucan is a major component of the *C. albicans* cell wall (19). Most β-1,3-glucan is located in the inner layer of the fungal cell wall and is therefore hidden from immune recognition by macrophages by the outer layer of mannan fibrils (20–22). Nevertheless, some β-1,3-glucan can become exposed at punctate foci on the lateral cell wall and at new bud scars on mother cells during cell division. These sites of β-1,3-glucan exposure serve as targets for immune recognition by macrophages and neutrophils (23), while monocytes recognize predominantly the mannan that shields β-1,3-glucan (24). In response to host inputs such as lactate or hypoxia, *C. albicans* cells induce the secretion of the β-glucanases Eng1 and Xog1 (25), and these enzymes are reported to remove exposed β-1,3-glucan from the fungal cell surface (25, 26). However, some phenotypic variability has been observed with respect to lactate-induced β-1,3-glucan masking and the contributions of glucanases to shaving (27, 28).

To clarify the terms used to describe these processes, “masking” has been defined as the decrease in β-1,3-glucan exposure on *C. albicans* cells observed experimentally after 5 h of growth in the presence of a masking signal, relative to control cells grown in the absence of that signal (9, 17). Meanwhile, “shielding” reflects the steric hindrance of β-1,3-glucan recognition by mannan fibrils in the outer layer of the *C. albicans* cell wall (20–22), and “shaving” represents the enzymatic removal of β-1,3-glucan that has become exposed at the cell surface (25, 26).

In this study, we reasoned that β-1,3-glucan masking is likely to be a dynamic phenotype that is dependent on growth rate (the rate at which new bud scars are generated) and shaving dynamics (the rate at which shaving enzymes are secreted, accumulate in the milieu, and remove exposed β-1,3-glucan). This dynamism might account for the reported variability in β-1,3-glucan masking (27, 28) and could potentially influence previous conclusions about genetic and environmental factors that influence the masking of this major immunoinflammatory PAMP (9, 17). We have tested these hypotheses in the present study.

Mathematical modeling has provided valuable and unexpected insights into the environmental responses of fungal pathogens (29–32) and host–fungus interactions (33–35). Therefore, we used an iterative cycle of modeling and experimentation to investigate the relative contributions of growth, β-1,3-glucan exposure, and shaving to the dynamics of lactate-induced β-1,3-glucan masking in *C. albicans*. Using this model, we have differentiated inputs that induce β-1,3-glucan shaving from those that simply retard growth or disrupt mannan shielding of β-1,3-glucan. We also define the relative contributions of signaling factors and the Eng1 and Xog1 β-glucanases to lactate-induced β-1,3-glucan shaving. Furthermore, we show that the model provides a platform for the investigation of other forms of PAMP masking that convert fungal cells into moving targets for the immune system.

## Results and Discussion

Our first objective was to develop a mathematical model that describes lactate-induced β-1,3-glucan masking in *C. albicans* to test our working hypothesis that a combination of growth/cell division together with the shaving of this PAMP is sufficient to account for the dynamics of β-1,3-glucan masking.

Regarding the impact of growth, our hypothesis was that β-1,3-glucan becomes exposed via the generation of new bud scars on mother cells following cell division (23, 36). The asymmetric expression of the Eng1 endoglucanase by daughter cells means that, unlike mother cells, the β-1,3-glucan exposure by daughters is minimized (26, 37). Exposed β-1,3-glucan is shaved off by secreted factors (25), which are reported to include the major exoglucanase Xog1, and the endoglucanase, Eng1 (25, 26). The secretion of these β-glucanases is induced by lactate (25), but we reasoned that their synthesis, secretion, and accumulation in the milieu must be time-dependent, which is bound to impact the dynamics of β-1,3-glucan shaving. Growth was assayed by monitoring OD_600_ over time, and β-1,3-glucan exposure was assayed by measuring the median fluorescence intensity (MFI) for Fc-dectin-1-stained *C. albicans* cells by flow cytometry at each timepoint (*Materials and Methods*). Two-step parameter estimation was used to quantify β-1,3-glucan shaving by these cells. First, growth was modeled as follows:

[1] N′(t)=μ(t)N(t),

[2] $$\mu \prime \left(t\right)=\mu (t)(\mu_{\max}-\mu (t)),$$

where $N\left(t\right)$ is the cell population density [$N\left(t\right)\propto$ OD_600_ (cell mass)], and $\mu_{max}$ is the maximum growth rate. Next, shaving was modeled:

[3] X′(t)=αN(t),

[4] B′(t)=ηN′(t)-β(t)X(t)B(t),

where X(t) is the quantity of shaving factors (e.g., enzymes) in the growth medium, synthesized and secreted by individual cells at a constant rate α;B(t) is the total β-1,3-glucan exposure; η denotes the average amount of β-1,3-glucan exposure per cell; and β(t) is the mass action coefficient for shaving enzymes acting on exposed β-1,3-glucan. We assumed $B\left(t\right)$ to be proportional to MFI (exposed β-1,3-glucan) and β(t) was taken to be a sigmoid increasing function of time (t), representing induction of the expression of shaving enzymes (see *Materials and Methods* for more details). Shaving activity was assumed to be proportional to the level of expressed enzyme:

σ(t)=αβ(t).

### Impact of Lactate Upon β-1,3-Glucan Exposure.

**Fig. 1.**
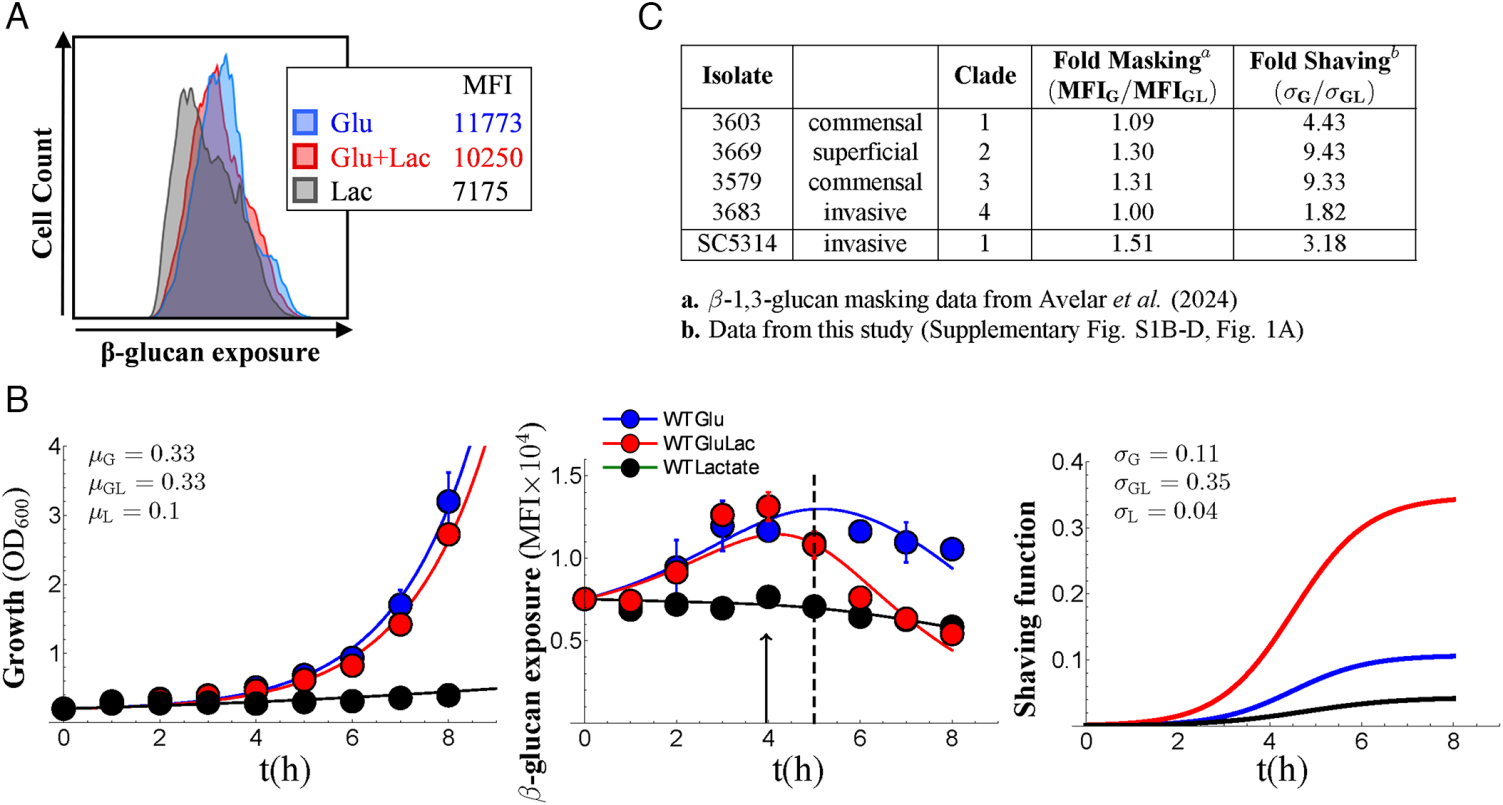
Dynamics of lactate-induced β-1,3-glucan shaving in *C. albicans.* (*A*) Quantification of β-1,3-glucan exposure by flow cytometry of Fc-dectin-stained cells. One experiment (of n = 3 for each timepoint) is shown for *C. albicans* SC5314 cultures grown for 5 h on glucose (SD, Glu; blue), glucose plus lactate (GluLac; red), or lactate as sole carbon source (Lac; black). The Median Fluorescence Intensity (MFI) for each population is indicated. (*B*) *Left*: Growth in SD without (blue) or with lactate (red), or in lactate (black). Symbols represent experimental data (means plus SD from triplicate assays). Lines of best fit as determined by the model are shown: growth rates on glucose (μ_G_), glucose plus lactate (μ_GL_), and lactate alone (μ_L_). *Middle*: dynamics of β-1,3-glucan exposure for *C. albicans* cells in the same cultures: the dotted line highlights the 5 h timepoint used previously to assay β-1,3-glucan masking; the arrow highlights the point of transition between cells grown on glucose or glucose plus lactate. *Right*: shaving functions (σ) determined by the model for cells in the same cultures. (*C*) Lactate-induced shaving in clinical isolates of *C. albicans* from different epidemiological clades. The fold-masking shown by these isolates in a previous study (27) [calculated by dividing the MFI on glucose (MFI_G_) by the MFI on glucose plus lactate (MFI_GL_)] is compared to the fold-change in shaving observed in the current study [calculated by dividing the shaving function for glucose plus lactate (σ_GL_) by the shaving function for glucose (σ_G_) using the data from (*A*)].

We first explored the influence of lactate on the dynamics of β-1,3-glucan exposure on the wild-type *C. albicans* clinical isolate SC5314 during growth on glucose. Cells were inoculated into SD (2% glucose, 0.65% yeast nitrogen base without amino acids) containing 0 or 2% lactate, and their relative growth rate (μ) and β-1,3-glucan exposure (MFI) were measured over an 8 h period (Fig. 1 *A* and *B*; *Materials and Methods*). Lactate treatment did not affect growth rate per se (Fig. 1*B*). Meanwhile, over this period, β-1,3-glucan exposure initially increased, consistent with the generation of new bud scars. However, MFI values for the lactate-treated samples and untreated controls diverged after 4 to 5 h: lactate-treated cells displayed a sharper decrease in β-1,3-glucan exposure. The simulations fitted the data with reasonable accuracy (Fig. 1*B*). Furthermore, the model estimated that glucose-grown cells display a low level of shaving and indicated that lactate induces an increase in β-1,3-glucan shaving (σ_GL_/σ_G_) of about threefold. This was entirely in keeping with reports that lactate induces β-1,3-glucan masking (9, 17).

These dynamics provided an explanation for the variability in lactate-induced β-1,3-glucan masking reported previously (27, 28). Masking was assayed by comparing β-1,3-glucan exposure after 5 h of growth with or without lactate (dotted line, Fig. 1*B*). This timepoint occurs close to the transition point at which lactate treatment starts to affect exposure (arrow, Fig. 1*B*). Therefore, subtle changes in growth (potentially mediated by differences in inoculum physiology or water, for example (27, 28) are likely to affect β-1,3-glucan masking. We tested this by reexamining four *C. albicans* clinical isolates from different epidemiological clusters (*SI Appendix*, Table S1). In a previous study, we reported that these isolates display limited lactate-induced β-1,3-glucan masking (27). However, more careful examination revealed that all four isolates do show robust lactate-induced β-1,3-glucan shaving (Fig. 1*C* and *SI Appendix*, Fig. S1). Therefore, while genetic differences between *C. albicans* strains clearly influence lactate-induced β-1,3-glucan masking (27), differences in growth probably account for much of the observed variability described previously (*SI Appendix*, Fig. S1) (27).

### Impact of Other Conditions Upon β-1,3-Glucan Exposure.

Strong β-1,3-glucan masking is induced when lactate serves as the primary carbon source, rather than as a supplement combined with glucose (9). The model allowed us to assess whether slow growth, strong shaving, or both underlie this masking. Growth of *C. albicans* SC5314 cells on lactate as sole carbon source was slow compared to growth on glucose and, consequently, no increase in β-1,3-glucan exposure was observed over the 8-h timecourse (Fig. 1*B*). As a result, after 5 h, the lactate-grown cells displayed twofold lower β-1,3-glucan exposure than the glucose-grown cells. This was consistent with our previous reports that growth on lactate induces strong β-1,3-glucan masking (9). However, minimal β-1,3-glucan shaving was predicted for cells growing on lactate alone (Fig. 1*B*). We conclude that the limited β-1,3-glucan exposure by lactate-grown cells is due to slow growth rather than strong shaving, and that true β-1,3-glucan shaving is stimulated by exposure to lactate, rather than growth on lactate.

**Fig. 2.**
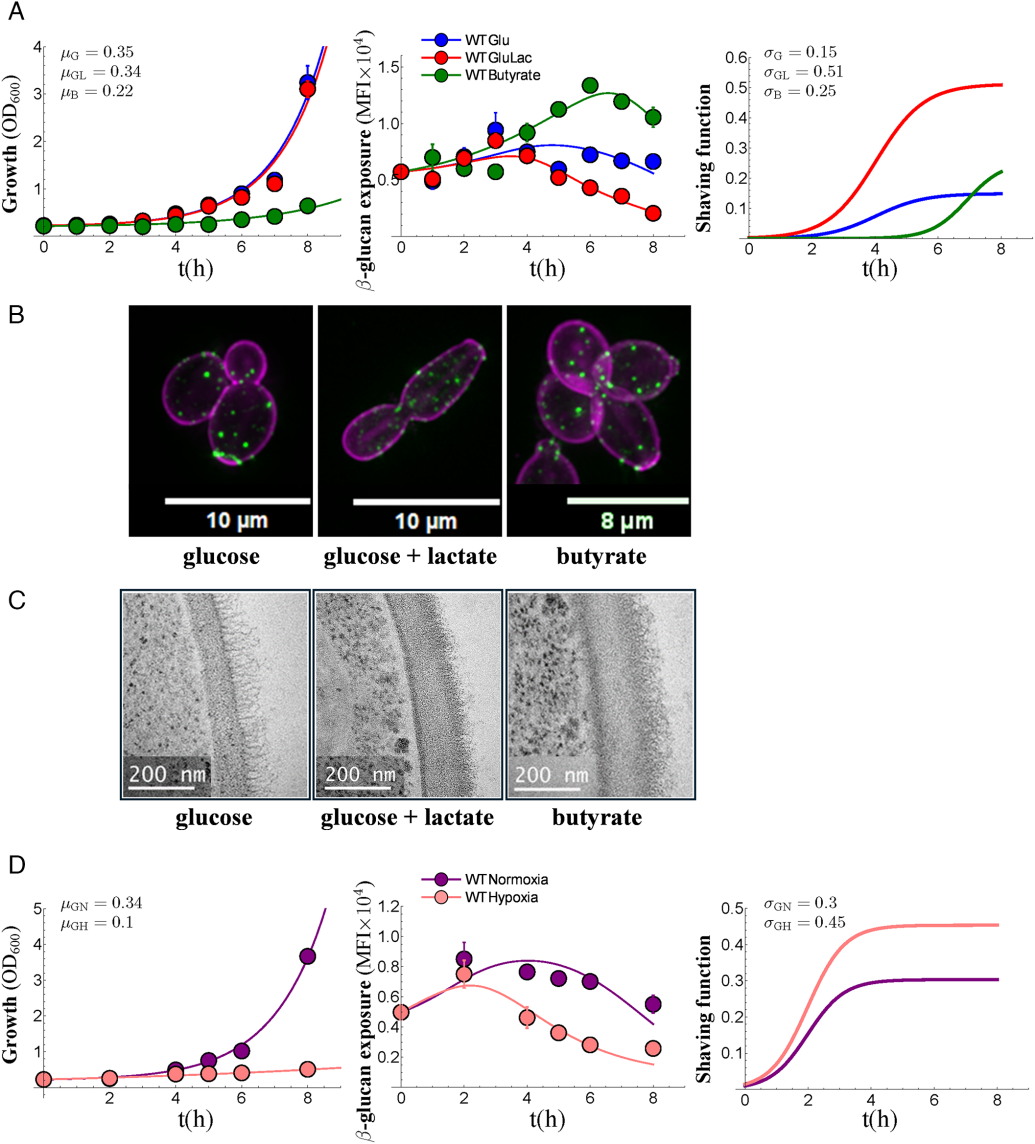
β-1,3-glucan shaving under other conditions. (*A*) Growth of *C. albicans* SC5314 on butyrate. *Left*: growth in SD without (Glu, blue, μ_G_) or with lactate (GluLac, red, μ_GL_), or in butyrate as sole carbon source (Butyrate, green, μ_B_). Experimental data (means plus SD from triplicate assays) are shown with the lines of best fit. *Middle*: dynamics of β-1,3-glucan exposure (MFI) for cells in the same cultures. *Right*: shaving functions (σ) for cells in the same cultures. Fluorescence microscopy (*B*) and TEM (*C*) of glucose-, glucose plus lactate-, and butyrate-grown cells. Scale bars and images are taken from the same micrographs. (*D*) Growth of *C. albicans* SC5314 on SD under hypoxia versus normoxia. *Left*: normoxic growth in SD (purple, μ_GN_), hypoxic growth in SD (pink, μ_GH_). Experimental data (means plus SD from triplicate assays) are shown with the lines of best fit. *Middle*: β-1,3-glucan exposure (MFI) by cells from the same cultures. *Right*: shaving functions (σ) for cells in the same cultures.

In contrast to lactate, growth on butyrate increases β-1,3-glucan exposure (17). Using the model, we tested whether this could be explained by effects on growth, shaving, or both. *C. albicans* SC5314 cells grew more slowly on butyrate than on glucose (Fig. 2*A*). Glucose-grown cells that were exposed to lactate displayed growth rates similar to those lacking lactate, but β-1,3-glucan exposure was reduced on the lactate-exposed cells and their shaving was elevated (Fig. 2*A*), thereby confirming our earlier findings (Fig. 1*B*). Despite their slow growth, after 4 h, the butyrate-grown cells started to expose more β-1,3-glucan than the glucose-grown controls (Fig. 2*A*). Interestingly, the model indicated that this was due to the delayed induction of shaving in butyrate-grown cells (Fig. 2*A*). Fluorescence microscopy revealed that the nature of the β-1,3-glucan exposure (at bud scars and punctate foci on the lateral cell wall) was similar for butyrate- and glucose-grown *C. albicans* cells (Fig. 2*B*). These findings are consistent with the idea that butyrate acts as an antagonist of Gpr1/Gpa2-mediated β-1,3-glucan masking (38). Transmission electron microscopy (TEM) revealed changes in the diameter of the inner cell wall layer for butyrate-grown cells, as well as for lactate-exposed cells (Fig. 2*C* and *SI Appendix*, Fig. S2). Therefore, butyrate-induced alterations in β-1,3-glucan exposure correlate with changes in cell wall architecture.

Hypoxia induces strong β-1,3-glucan masking (16). Therefore, we tested whether this is because hypoxia influences growth, shaving, or both. Here, we grew *C. albicans* SC5314 cells on glucose (SD) under hypoxic conditions and compared them with normoxic SD controls (*Materials and Methods*). The hypoxic cells grew more slowly and exposed less β-1,3-glucan (Fig. 2*D*), and they have thinner cell walls (16). Shaving was estimated to be higher in hypoxic than normoxic cells (Fig. 2*D*), suggesting that the strong β-1,3-glucan masking observed under hypoxic conditions (16) is due to a combination of slower growth and increased shaving.

Taken together, these experiments confirm that, while growth rate does influence β-1,3-glucan exposure, there is no strict correlation between growth and β-1,3-glucan exposure in *C. albicans* (27). For example, while growth is relatively slow on lactate, hypoxia, or butyrate, the first two conditions lead to reduced β-1,3-glucan exposure, while the latter condition enhances exposure (Figs. 1 and 2).

### Regulation of β-1,3-Glucan Masking.

Lactate-induced β-1, 3-glucan masking is thought to be activated via Gpr1/Gpa2 and protein kinase A (PKA) signaling (9, 17). Gpr1 is a G-protein-coupled receptor (GPCR) that acts in concert with the G-protein α subunit, Gpa2 (39). Gpr1 lies upstream on the PKA signaling pathway (39) and might act as a lactate receptor because it displays sequence similarity to the mammalian lactate receptor (9). *TPK1* and *TPK2* encode partially redundant catalytic subunits of PKA (40, 41). β-1,3-glucan masking is blocked in *C. albicans gpr1 gpa2* and in *tpk1 tpk2* double mutants (9, 17), but it is not clear from whether this is due to defects in growth or shaving.

**Fig. 3.**
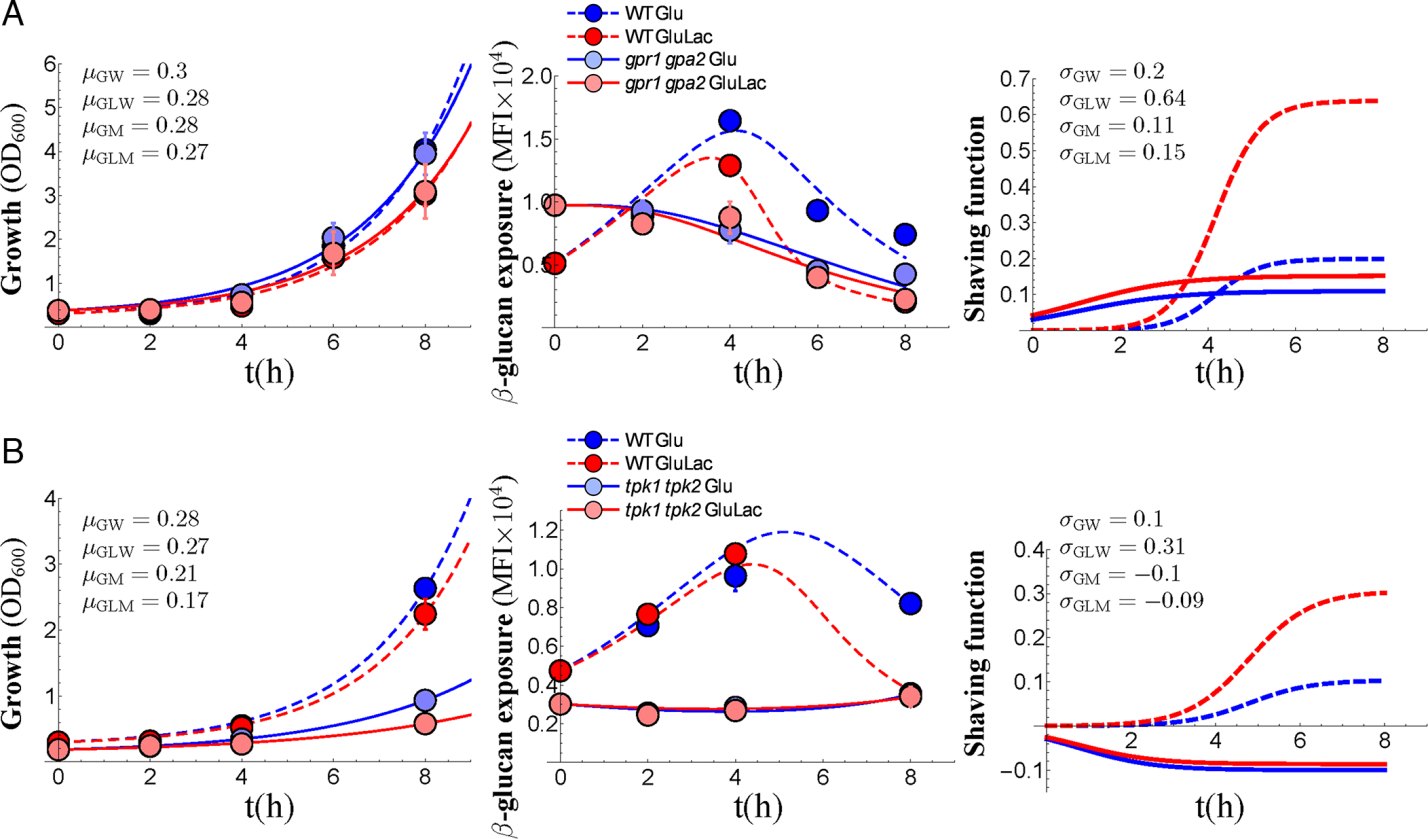
Inactivation of Gpr1/Gpa2 and PKA signaling inhibits β-1,3-glucan shaving. Dynamics of growth and β-1,3-glucan shaving for *C. albicans* strains grown in SD with or without lactate: (*A*) *gpr1 gpa2* cells versus wild type parent (Ca372); (*B*) *tpk1 tpk2* cells versus wild type parent (SN152) (*SI Appendix*, Table S1). *Left*: comparison of growth of wild type in SD without lactate (Glu, dark blue, μ_GW_) or with lactate (GluLac, red, μ_GLW_), with the mutants in SD without lactate (light blue, μ_GM_) or with lactate (pink, μ_GLM_). Means plus SD from triplicate assays are shown with the lines of best fit: wild type, dotted lines; mutants, solid lines. *Middle*: dynamics of β-1,3-glucan exposure (MFI) for cells in the same cultures. *Right*: shaving functions (σ) for cells in the same cultures: wild type, dotted lines; mutants, solid lines.

We compared the dynamics of lactate-induced β-1,3-glucan masking in *C. albicans gpr1 gpa2* cells with their isogenic wild-type control Ca372, which is derived from SC5314 (*SI Appendix*, Table S1). Like SC5314 (Fig. 1), Ca372 responded to lactate by enhancing β-1,3-glucan shaving about threefold (Fig. 3*A*). The *gpr1 gpa2* mutant grew at similar rates to its wild type control (Ca372) but displayed no increase in β-1,3-glucan exposure during growth. This could be taken to imply that *gpr1 gpa2* cells are hyper-shavers, as Gpr1–Gpa2 have been suggested to promote β-1,3-glucan exposure (42). However, our dynamical analyses revealed that *C. albicans gpr1 gpa2* cells display higher levels of β-1,3-glucan exposure at time zero, and lower levels of shaving than wild-type cells (Fig. 3*A*). Significantly, β-1,3-glucan shaving was not induced by lactate in *gpr1 gpa2* cells (Fig. 3*A*). Hence, we favor the idea that Gpr1–Gpa2 signaling is required for lactate-induced β-1,3-glucan shaving.

As reported previously (41), the *C. albicans tpk1 tpk2* mutant grew more slowly than its wild type parent, SN152 (Fig. 3*B*), which is also derived from SC5314 (*SI Appendix*, Table S1). Not surprisingly, therefore, *tpk1 tpk2* cells exposed less β-1,3-glucan than their wild type control and yet no decrease in β-1,3-glucan exposure was observed over the timecourse (Fig. 3*B*). No differences in shaving were observed for *tpk1 tpk2* cells in the presence or absence of lactate. The dependence of lactate-induced β-1,3-glucan shaving upon PKA signaling was then confirmed in epidemiologically divergent *C. albicans* clinical isolates from different niches using the inhibitors MDL12330 and myristoylated PKI (43) (*SI Appendix*, Fig. S3). The negative shaving (σ) values observed for *tpk1 tpk2* cells (Fig. 3*B*) were consistent with an altered cell wall architecture, as reported previously (41, 44), but could also be explained, for example, by increased degradation of shaving enzymes (*Materials and Methods*). Meanwhile, as before (Figs. 1 and 3*A*), wild-type control cells displayed a threefold increase in β-1,3-glucan shaving in response to lactate (Fig. 3*B*). Taken together, the data show that lactate-induced β-1,3-glucan shaving is dependent on PKA signaling.

### Mechanisms Underlying Lactate-Induced β-1,3-Glucan Masking.

**Fig. 4.**
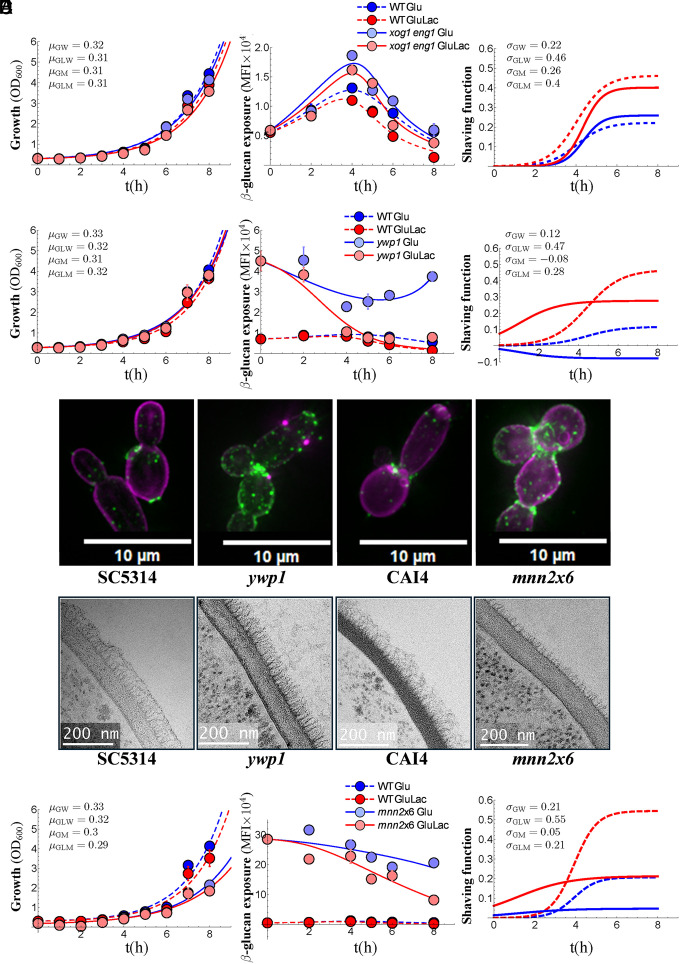
Mechanisms underlying β-1,3-glucan shaving and masking. Growth and β-1,3-glucan shaving for glucanase-defective *C. albicans* strains and mutants with cell wall defects. *Left*: growth of wild type controls (dark colors) and mutants (light colors) without lactate (Glu, dark/light blue, μ_GW_, μ_GM_) and with lactate (GluLac, red/pink, μ_GLW_, μ_GLM_). Means plus SD from triplicate assays are shown with the lines of best fit: wild type, dotted lines; mutants, solid lines. *Middle*: dynamics of β-1,3-glucan exposure (MFI) for cells in the same cultures. *Right*: shaving functions (σ) for cells in the same cultures: wild type, dotted lines; mutants, solid lines. (*A*) Growth, β-1,3-glucan exposure and shaving for four independent *xog1 eng1* double mutants versus wild type controls: WT A03, WT A08, WT A10, WT A11, *xog1 eng1* B07a, *xog1 eng1* B07b, *xog1 eng1* B11, *xog1 eng1* C10. (*B*) Growth and β-1,3-glucan shaving for three independent *ywp1* strains versus the wild type control, SC5314. Fluorescence microscopy (*C*) and TEM (*D*) of *C. albicans ywp1* and *mnn2*x6 cells and their parental wild-type controls, SC5314 and CAI4, respectively. Each scale bar is drawn from the same micrograph as the corresponding image. (*E*) Analysis of growth and β-1,3-glucan shaving for *C. albicans mnn2*x6 cells: wild type, Ca372; *mnn2*x6, NGY600. Strains are described in *SI Appendix*, Table S1.

Secreted factors promote β-1,3-glucan shaving in *C. albicans* (25). These include the endoglucanase Eng1 (26) and possibly the exoglucanase Xog1 (25, 28), both of which are induced in the secretome in a manner dependent upon PKA signaling (25). First, using high-pressure ion chromatography (HPIC) to examine the carbohydrate content of the cell wall, we confirmed our prediction (28) that the loss of Eng1 or Xog1 does not significantly affect the overall glucan content of the *C. albicans* cell wall (*SI Appendix*, Fig. S4). We then compared the dynamics of β-1,3-glucan exposure in independent *xog1, eng1,* and wild-type control strains (*SI Appendix*, Table S1). The inactivation of *XOG1* or *ENG1* did not affect growth or the normal biphasic pattern of β-1,3-glucan exposure over the course of these experiments (*SI Appendix*, Fig. S5). Furthermore, the *xog1* and *eng1* mutants retained lactate-induced β-1,3-glucan shaving (*SI Appendix*, Fig. S5), consistent with the idea that these secreted glucanases contribute to, but are not essential for β-1,3-glucan shaving (26, 28). To test for possible compensatory effects following the deletion of *XOG1* or *ENG1,* we examined the behavior of independent *xog1 eng1* double mutants. No clear evidence for compensatory effects was observed, as four *xog1 eng1* mutants retained similar levels of lactate-induced β-1,3-glucan shaving to their wild-type controls (Fig. 4*A*). However, the double mutants did expose more β-1,3-glucan than the wild type controls (Fig. 4*A*), further supporting the idea that Eng1 and Xog1 glucanases contribute to shaving (25, 26, 28).

Most β-1,3-glucan in the *C. albicans* cell wall is shielded from immune recognition by the outer layer of lengthy mannan fibrils (19, 20). Hence, lactate-induced β-1,3-glucan masking might be mediated, at least in part, by adjustments to the outer cell wall layer. The yeast wall protein Ywp1 appears to promote β-1,3-glucan shielding, because *ywp1* cells are reported to display enhanced β-1,3-glucan exposure (26, 45). Therefore, we constructed three independent *ywp1* mutants (*Materials and Methods*) and examined their cell walls. Fluorescence microscopy of Fc-dectin-1-stained cells showed that, like wild type cells, *ywp1* cells exposed β-1, 3-glucan at bud scars and punctate foci on the lateral cell wall (Fig. 4*B*). Further analysis by HPIC revealed that the walls of *ywp1* cells contained significantly more glucan and chitin and less mannan than the wild-type controls (*SI Appendix*, Fig. S4). TEM revealed that this difference was reflected in a thinner outer mannan layer and a thicker inner glucan layer in *ywp1* cell walls compared to their wild type controls (Fig. 4*C* and *SI Appendix*, Fig. S2). This was consistent with the relatively high levels of β-1,3-glucan exposure reported for *ywp1* mutants (26, 45) and our observation that starting *ywp1* inocula exposed over fourfold more β-1,3-glucan than their wild type controls (Fig. 4*B*). Significantly, the *ywp1* mutants responded to lactate by strongly inducing β-1,3-glucan shaving (σ_Glu_ = −0.08 compared to σ_GluLac_ = 0.28: Fig. 4*B*). Therefore, lactate-induced β-1,3-glucan masking is not dependent upon Ywp1.

We further explored the role of mannan fibrils in β-1,3-glucan shielding using an *mnn2*x6 mutant that lacks all six members of the *MNN2* gene family of mannosyltransferases that add α1,2-mannose side chains to the α 1,6-mannose backbone, thereby forming the *N*-mannan outer chain branches. The total outer mannan layer of the cell wall is severely compromised in this mutant (46). This was confirmed by HPIC which revealed dramatically reduced mannan content (*SI Appendix*, Fig. S4) and by TEM showing a greatly reduced outer mannan layer compared to the wild-type control under our experimental conditions (Fig. 4*D* and *SI Appendix*, Fig. S2). As expected given this reduction in mannan shielding, *mnn2*x6 cells displayed extremely high levels of β-1,3-glucan exposure: about 25-fold higher than wild-type cells in the starting inocula (Fig. 4*E*). Despite their high levels of β-1,3-glucan exposure, *mnn2*x6 cells still showed a circa fivefold increase in β-1,3-glucan shaving in response to lactate (Fig. 4*E*). This confirmed that lactate-induced β-1,3-glucan shaving is not dependent on mannan shielding.

Mannan shielding was dramatically reduced in *mnn2*x6 cells (Fig. 4*D*). Yet, their β-1,3-glucan exposure was largely confined to bud scars and punctate foci, similar to wild type cells (Fig. 4*C*) and was not distributed evenly across the lateral cell wall. This has important implications for the nature of β-1,3-glucan exposure in *C. albicans*. Dectin-1 binds β-1,3-gluco-oligomers of 11-13 residues long (47). However, most β-1,3-glucan in the yeast cell wall exists in a triple helical conformation, which limits immune recognition (48, 49) and, consequently, most β-1,3-glucan appears to remain “invisible” to dectin-1 even in *mnn2*x6 cells (Fig. 4*C*). Therefore, exposure, as defined by dectin-1 recognition, may occur at frayed ends of glucan triple helices where a filament(s) of single-stranded β-1,3-glucan emerges at the cell surface. This would account for the punctate nature of β-1,3-glucan exposure on the *mnn2*x6 cell wall.

## Conclusions

Our multidisciplinary combination of mathematical modeling and experimentation indicates that changes in growth (cell division) and β-1,3-glucan shaving underlie the dynamics of β-1,3-glucan masking in *C. albicans*. This is valid for a range of masking stimuli against a range of myeloid cells with the exception of monocytes. The model has also revealed why the lactate-induced β-1,3-glucan masking phenotype, which historically has been measured at a fixed timepoint (16, 17, 27, 28), has proven sensitive to factors that affect *C. albicans* growth and physiology (28). Furthermore, our data show that *C. albicans* strains isolated from different types of infection and from different epidemiological clades display robust lactate-induced β-1,3-glucan shaving.

Our analyses have also highlighted the importance of GPCR-PKA signaling for the activation of lactate-induced β-1,3-glucan masking in *C. albicans*, and established the significance of major secreted glucanases in β-1,3-glucan shaving. Furthermore, our examination of shielding has provided important insights into the nature of β-1,3-glucan exposing features at the *C. albicans* cell surface. Even when the outer layer of mannan fibrils is severely compromised, only certain features are recognized by Fc-dectin-1: Bud scars and punctate foci on the lateral cell wall. Each of these foci might represent single-stranded fibers that occasionally extend, from the end of a β-1,3-glucan triple helix, above the fungal cell surface into the milieu to become accessible to this critical pattern recognition receptor.

These findings are significant in the context of fungal colonization, infection, and antifungal immunity. β-1,3-glucan masking represents an immune evasion strategy that is activated during fungal adaptation to host niches to convert *C. albicans* into a moving target for the immune system (8). The fungus has evolved the ability to activate β-1,3-glucan masking in response to specific host signals such as lactate, hypoxia, ambient pH, and iron depletion (9, 10, 16, 17). These responses are anticipatory: They are activated even before the fungus is exposed to attack by innate immune cells. Hence, β-1,3-glucan masking is thought to represent a form of adaptive prediction (50) that enhances the fitness of *C. albicans* in certain host niches (8). Indeed, β-1,3-glucan masking appears to enhance colonization of the large intestine (38) and attenuates immune responses against the fungus in vitro and in vivo (9, 16–18). Significantly, this type of immune evasion strategy has been observed in other pathogenic *Candida* species (16) and in evolutionarily distant fungal pathogens, such as *Histoplasma capsulatum* (51). Accordingly, our multidisciplinary approach to defining the dynamics of PAMP exposure may prove useful to understand host–microbe interactions in other major fungal pathogens.

## Materials and Methods

### Mathematical Model of Growth.

The mathematical model aims to elucidate the dynamics of β-1,3-glucan exposure on *C. albicans* yeast cell walls, specifically through the processes of cell growth and enzymatic shaving. The behavior of the system is described by a set of ordinary differential equations (ODEs).

To model *C. albicans* growth, the change in the cell population density $N\left(t\right)$ over time t is given by the following equation:

[5] $$\frac{\mathrm{dN}}{\mathrm{dt}}=\mu \left(t\right)N\left(t\right),$$

[6] $$\frac{d\mu }{\mathrm{dt}}=\mu \left(t\right)(\mu_{\max}-\mu \left(t\right)).$$

The latter equation represents an increase in time t of the relative growth rate $\mu \left(t\right)$ from an initial value of $\mu \left(0\right)=\mu_{0}$ to an eventual value of $\lim_{t\to \infty }\mu {=\mu }_{max}$ as explained in ref. 52.

The analytical solutions to these ODEs [**1**] and [**2**] are as follows:

[7] $$N\left(t\right)=N_{0}(1+\frac{\mu_{0}}{\mu_{\max}}(e^{\mu_{\max}t}-1)),$$

[8] $$\mu \left(t\right)=\frac{\mu_{\max}e^{\mu_{\max}t}}{e^{\mu_{\max}t}-1+\mu_{\max}/\mu_{0}}.$$

where $N\left(0\right)=N_{0}$ is the initial cell population density.

The parameters $N_{0},\mu_{0},$ and $\mu_{max}$ were estimated by fitting Eq. **3** to the experimental growth data (OD_600_). Numerical results were obtained via simulations performed by means of a stand-alone server application written in Java 17. We adapted a gradient search method (53) to determine optimal parameters $\mu_{0}$ and $\mu_{max}$ which satisfy Eq. **3** and to fit the experimental data. To demonstrate that these values are statistically significant, we calculated *P*-values and showed they are less than 0.05 (*SI Appendix*, Table S2).

### Mathematical Model of β-1,3-Glucan Exposure.

The MFI for a population of dectin-1-stained *C. albicans* cells was measured by flow cytometry (below) at each time point t. This measurement was divided by 10^4^ and then multiplied by the total number of cells $N\left(t\right)$ at each time t to give the total exposed β-1,3-glucan at time t (in MFI units), $B\left(t\right)$.

Under reference conditions, where no shaving occurs, we assumed

[9] B(t)=ηN(t),

where η is the average amount of β-1,3-glucan exposure per cell, assumed to be a constant.

Under shaving-inducing conditions, we assumed that the appearance of β-1,3-glucan is unaltered as a function of increasing cell numbers, but that there is now also a disappearance term which we assumed to be proportional to both $B\left(t\right)$ and some factor X, presumed to be one or more enzymes (for example, the secreted β-glucanases Eng1 and Xog1 (25, 26, 28), and we are assuming a mass action term. If *C. albicans* cells synthesize and secrete these factors at a constant rate α per cell from time t=0 onward, then X will itself be proportional to $\int_{0}^{t}N\left(\tau \right)d\tau$. These assumptions lead to the following equations:

[10] $$\frac{\mathrm{dX}}{\mathrm{dt}}=\alpha N(t),$$

[11] $$\frac{\mathrm{dB}}{\mathrm{dt}}=\eta \frac{\mathrm{dN}}{\mathrm{dt}}-\beta \left(t\right)X\left(t\right)B(t),$$

with initial conditions $X\left(0\right)=0$ and $B\left(0\right)=B_{0}$ and where

[12] $$\beta \left(t\right)=\frac{\beta }{1+e^{s(t_{s}-t)}}$$

is the time-varying coefficient for the mass action interaction between the shaving enzymes and the exposed β-1,3-glucan, with the parameters s and $t_{s}$ corresponding to the speed and timing of shaving, respectively.

We substitute the solution of ODE [**6**], which is $X\left(t\right)=\alpha \int_{0}^{t}N\left(\tau \right)d\tau$, into ODE [**7**] to obtain a linear ODE with varying coefficients, giving

[13] $$\frac{\mathrm{dB}}{\mathrm{dt}}=\eta \frac{\mathrm{dN}}{\mathrm{dt}}-\sigma (t)\int_{0}^{t}N\left(\tau \right)d\tau B(t),$$

where we set $\sigma \left(t\right)=\alpha \beta \left(t\right)$, which we call hereafter a shaving function.

The parameters η, s, and $t_{s}$ were estimated by numerically solving Eq. **9** and subsequently computing the ratio $B\left(t\right)/N\left(t\right)$ and subsequently fitting it to the experimental data on β-1,3-glucan exposure per cell (MFI/10^4^). Numerical results were obtained via simulations performed by means of a stand-alone server application written in Java 17. We used the Gear implicit fourth-order method (54) to calculate a numerical solution of Eq. **9**. We adapted a gradient search method (53) for determining best-fitting parameters η, s, and $t_{s}$ which satisfy Eq. **9** and fit the experimental data. We obtained the optimal parameter values by fitting the model to the experimental data. Their *P*-values were less than 0.05, demonstrating that the obtained parameter values are statistically significant (*SI Appendix*, Table S2).

For each set of shaving-inducing conditions, we obtain a particular shaving function $\sigma \left(t\right)$, and we directly compare these functions between conditions.

Besides a production term, with X being created and secreted in proportion to N, we allow for the possibility of a loss term that is also proportional to N, which represents a breakdown or inactivation process associated with the cell mass. In either case, we then have

$$\alpha =\alpha^{+}-\alpha^{-},$$

with both $\alpha^{+}$ and $\alpha^{-}$ positive rate constants. Here, $\alpha^{+}$ represents the production of factor X, whereas $\alpha^{-}$ represents the destruction of X by some process, for example, via secreted proteinases. Under these assumptions, it is possible to obtain negative α values, which would imply negative σ values, since $\sigma \left(t\right)=\alpha \beta \left(t\right)$, and $\beta \left(t\right)$ is nonnegative by definition [**8**].

### Fungal Strains and Growth.

The *C. albicans* isolates and mutants used in this study are described in *SI Appendix*, Table S1. Three independent homozygous *ywp1* null mutants were made in *C. albicans* SC5314 using CRISPR-Cas9 and the *SAT1* marker, as described previously (28, 55). Four independent *C. albicans xog1 eng1* double mutants were made by deleting *ENG1* in the *xog1* strains Ca2609, Ca2613, and Ca2615 (*SI Appendix*, Table S1) using CRISPR-Cas9, selecting for the *CaHygB* marker on YPD (2% glucose, 1% yeast extract, 2% myco-peptone) containing 600 μg/ml hygromycin plus the adjuvant, 1 mg/ml sodium molybdate (28, 55, 56). Loss of the relevant open reading frame and the concomitant insertion of the relevant marker were confirmed in each mutant by diagnostic PCR (28).

Fresh *C. albicans* colonies (less than one week old) from YPD plates were used to inoculate starter cultures in glucose-containing minimal SD medium (2% glucose, 0.65% yeast nitrogen base without amino acids) (57). These were grown at 30 °C at 200 rpm for about 15 h (28). Then, on the morning of the experiment, these starter cultures were used to inoculate fresh 5 mL cultures of the specified medium (starting OD_600_ = 0.2), grown at 30 °C at 200 rpm, and harvested for analysis at the specified timepoints over an 8-h period. These media were 0.65% yeast nitrogen base without amino acids containing 2% glucose (SD normoxic control); 2% glucose plus 2% lactate (lactate exposure); 2% lactate (lactate as sole carbon source); 2% butyrate (butyrate as sole carbon source); and 2% glucose (hypoxic: degassed, under nitrogen) (16, 17). Growth (OD_600_) was monitored hourly. The involvement of PKA signaling in clinical isolates was confirmed using the inhibitors MDL12330 (417 μM) and myristoylated PKI (25 μM) (43).

### β-1,3-Glucan Exposure.

β-1,3-glucan exposure was quantified by flow cytometry, as described previously (17, 25, 28). Briefly, *C. albicans* cells were grown in SD, as described above, fixed with 50 mM thimerosal (Sigma-Aldrich, Dorset, UK), and then stained with Fc-dectin-1 and anti-human IgG linked to Alexafluor 488 (Jackson ImmunoResearch, Ely, UK). An Attune NxT flow cytometer was used to measure fluorescence intensities for 10,000 cells per sample. The median fluorescence intensity (MFI) for each sample was determined using FlowJo v.10 software. Means and SD were calculated from three independent biological replicates. Each mutant was compared with their isogenic “wild type” parent.

β-1,3-glucan exposing features at the cell surface were examined by confocal fluorescence microscopy (28). *C. albicans* cells were grown and fixed with thimerosal, as described above. These cells were stained with Fc-dectin-1 and IgG-AF488 (β-1,3-glucan) and ConA-AF647 (mannan), and then imaged using a DeltaVision Elite fluorescence microscope (100× objective, 1.40 numerical aperture) capturing 10 µm Z-stacks of 50 images with a pco.edge sCMOS camera. Following deconvolution of 3D stacks to remove out-of-focus light, 2D images were generated via ImageJ (version 1.54).

### Cell Wall Analyses.

Cell wall architecture was examined by TEM high high-pressure freezing as previously reported (28), and freeze-substitution using a Leica AFS2 (Leica Microsystems, Milton Keynes, UK). *C. albicans* strains were grown in SD, as described above and rapidly subjected to high-pressure freezing (58). Samples were transferred to 2% osmium in acetone and embedded in Epon (28). Ultrathin sections (60 nm) were cut, lead citrate was used for contrast, and imaged at ×100K magnification using a JEOL 1400 JEM transmission electron microscope with a digital camera (ES1000W, Gatan, Ametek, Abingdon, UK). At least 30 *C. albicans* cells were imaged per condition. Sections of their cell walls were selected at random, and the diameters of the inner and outer layers were measured (10 measurements per cell) using the line tool in ImageJ (version 1.54).

Cell wall carbohydrates were assayed by high-pressure ion chromatography (HPIC) using established procedures (59, 60) with the following modifications. Cell wall hydrolysates (0.4 µL) were analyzed using a Dionex ICS-4000 (Thermo Scientific) equipped with a CarboPac PA20 column (0.4 × 150 mm), guard column, and an ED50 Pulsed amperometric detector (PAD). Samples were eluted with an isocratic gradient of 5 mM KOH at a flow rate of 0.008 mL/min. The column was washed with 100 mM KOH and then equilibrated with 5 mM KOH before running the next sample.

### Statistical Analyses.

These were performed in GraphPad Prism 10. Data were generated using at least three independent biological replicates, are expressed as means ± SD, and were analyzed statistically using the tests specified. The following *P*-values were considered: not significant > 0.05; *P < 0.05; **P <0.01; ***P < 0.001; ****P < 0.0001.

## Supplementary Material

Appendix 01 (PDF)

## Data Availability

All study data are included in the article and/or *SI Appendix*.
