# Supplementary material for: Protein kinase A signaling regulates immune evasion by shaving and concealing fungal β-1,3-glucan
Source: Proc Natl Acad Sci U S A. 2025 Jun 9;122(24):e2423864122. doi: 10.1073/pnas.2423864122 (PMC12184398; doi:10.1073/pnas.2423864122)
Supplement: Supplementary file 1 — Appendix 01 (PDF) [file pnas.2423864122.sapp.pdf]

PNAS

Supporting Information for
Protein kinase A signalling regulates immune evasion by shaving and concealing fungal β-1,3-glucan.

Arnab Pradhan, Olga A. Nev, Ian Leaves, Oleg A. Nev, Qinxi Ma, Gillian Milne, Grace Patterson, Mihai G. Netea, Lars P. Erwig, Rhys A. Farrer, Gordon D. Brown, Hugo A. van den Berg, Neil A.R. Gow and Alistair J.P. Brown

Corresponding author: Alistair J.P. Brown

Email: a.j.p.brown@exeter.ac.uk

This PDF file includes:

Figure S1. Lactate-induced β-1,3-glucan shaving in *C. albicans* clinical isolates from different epidemiological clusters.

Figure S2. Diameter of the cell wall under different conditions.

Figure S3. PKA signalling is required for lactate-induced β-1,3-glucan shaving in divergent clinical isolates of *C. albicans.*

Figure S4. Impact of mutations on the carbohydrate content of the *C. albicans* cell wall.

Figure S5. Influence of Xog1 and Eng1 β-1,3-glucanases on shaving.

Table S1. Strains used in this study.

Table S2. Optimal parameters values obtained by fitting the model (1) – (4) from the main text to the experimental data on growth and β-1,3-glucan exposure.

Supplementary Figure S1

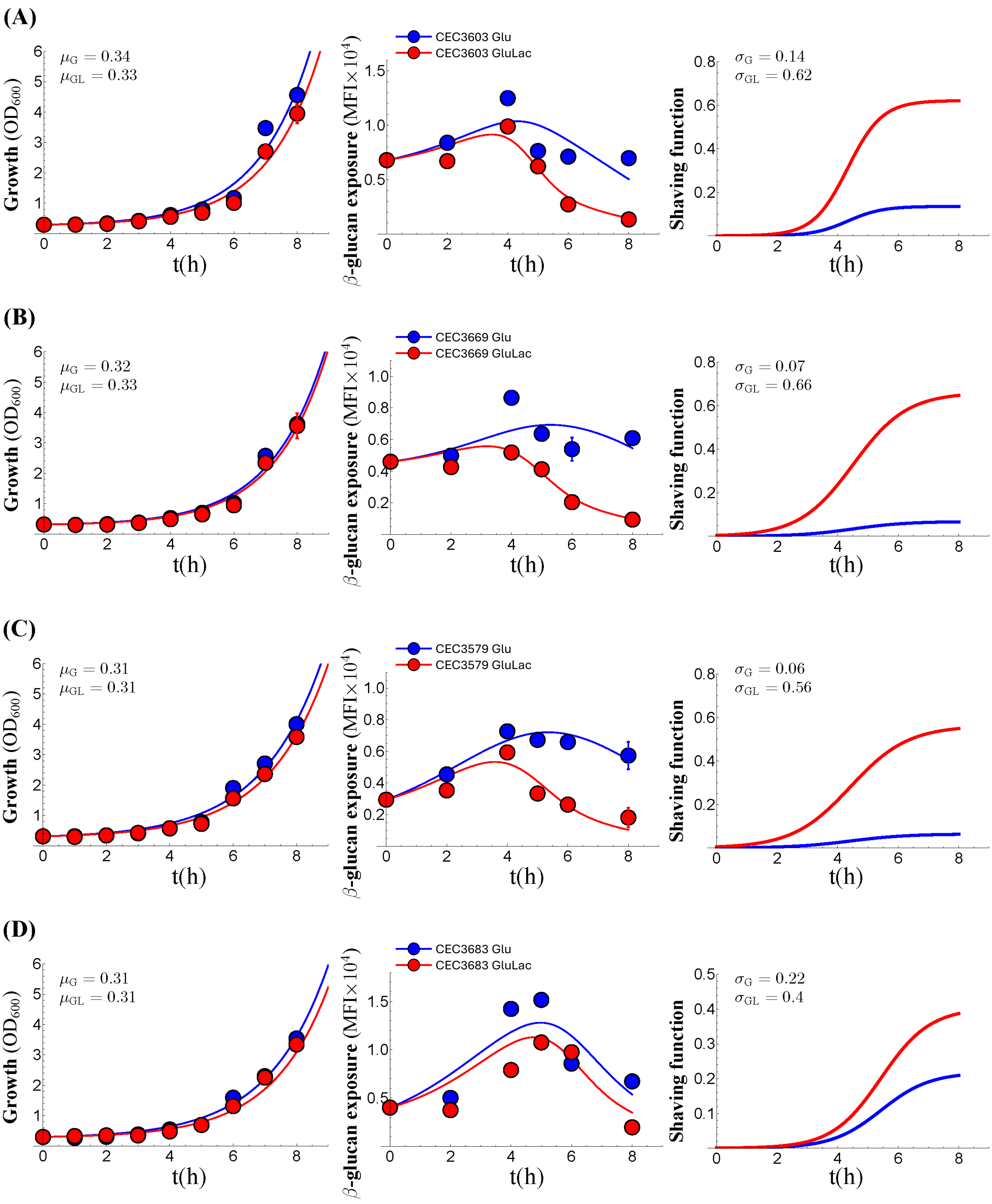

Fig. S1. Lactate-induced β-1,3-glucan shaving in *C. albicans* clinical isolates from different epidemiological clusters.

In a previous screen, *C. albicans* clinical isolates displayed significant variability in their lactate-induced β-1,3-glucan masking [Avelar GM*, et al* (2024) *mBio* 15: e0189823-23]. This cytometric screen compared β-1,3-glucan exposure in the presence and absence of lactate at a single five-hour timepoint and was performed in 96-well titre plates. Differences in growth between isolates under these conditions could conceivably have contributed to the observed variability in β-1,3-glucan masking [Avelar GM*, et al* (2024) *mBio* 15: e0189823-23]. Therefore, four isolates from different epidemiological clades that had displayed limited lactate-induced β-1,3-glucan masking in this screen (Table S1) were selected for more detailed analysis. The dynamics of growth (OD_{600}) and β-1,3-glucan exposure (MFI) for these isolates were examined over an eight-hour timecourse, and the impact of lactate treatment upon their shaving (σ) calculated: **(A)** CEC3603; **(B)** CEC3669; **(C)** CEC3579; **(D)** CEC3683. Means and standard deviations from three independent experiments are presented along with the lines of best fit generated using the model. The lactate-induced changes in β-1,3-glucan shaving are compared to the fold masking reported previously for these isolates in Fig. 1B. These data confirm that all four clinical isolates do display robust lactate-induced β-1,3-glucan shaving.

Supplementary Figure S2

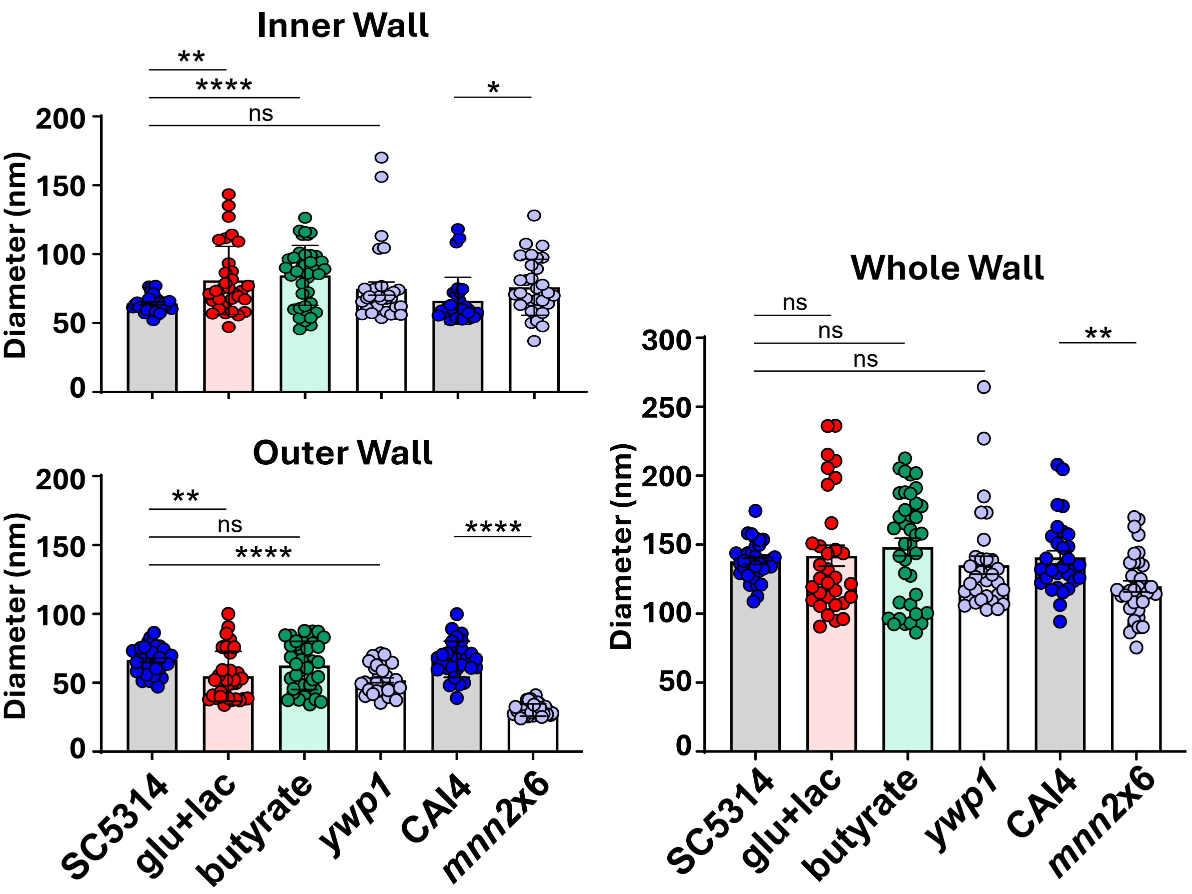

Fig. S2. Diameter of the cell wall under different conditions.
C. albicans cells were subjected to TEM, and the diameters of the inner and outer layers of their cell walls (left panels) as well as the diameters of their whole cell walls (right panel) were measured from TEM sections using the line tool in ImageJ (Materials and Methods). *C. albicans* SC5314 cells were grown in minimal medium containing glucose plus lactate (glu+lac; red), or butyrate (green) and compared to the control (the same strain grown in glucose, SD medium; dark blue). Also, *C. albicans ywp1* and *mnn2*x6 mutants (pale blue) were grown on SD medium and compared to their wild type controls, SC5314 and CAI4, respectively (dark blue). The data represent means and standard deviations from $n \geq 30$ cells (10 measurements per cell) and were analysed using Brown-Forsythe and Welch ANOVA (for comparisons with SC5314) or Mann-Whitney tests (for comparison of *mnn2*x6 with CAI4): ns, not significant; * $p < 0.05$; ** $p < 0.01$; *** $p < 0.001$; **** $p < 0.0001$..

Supplementary Figure S3

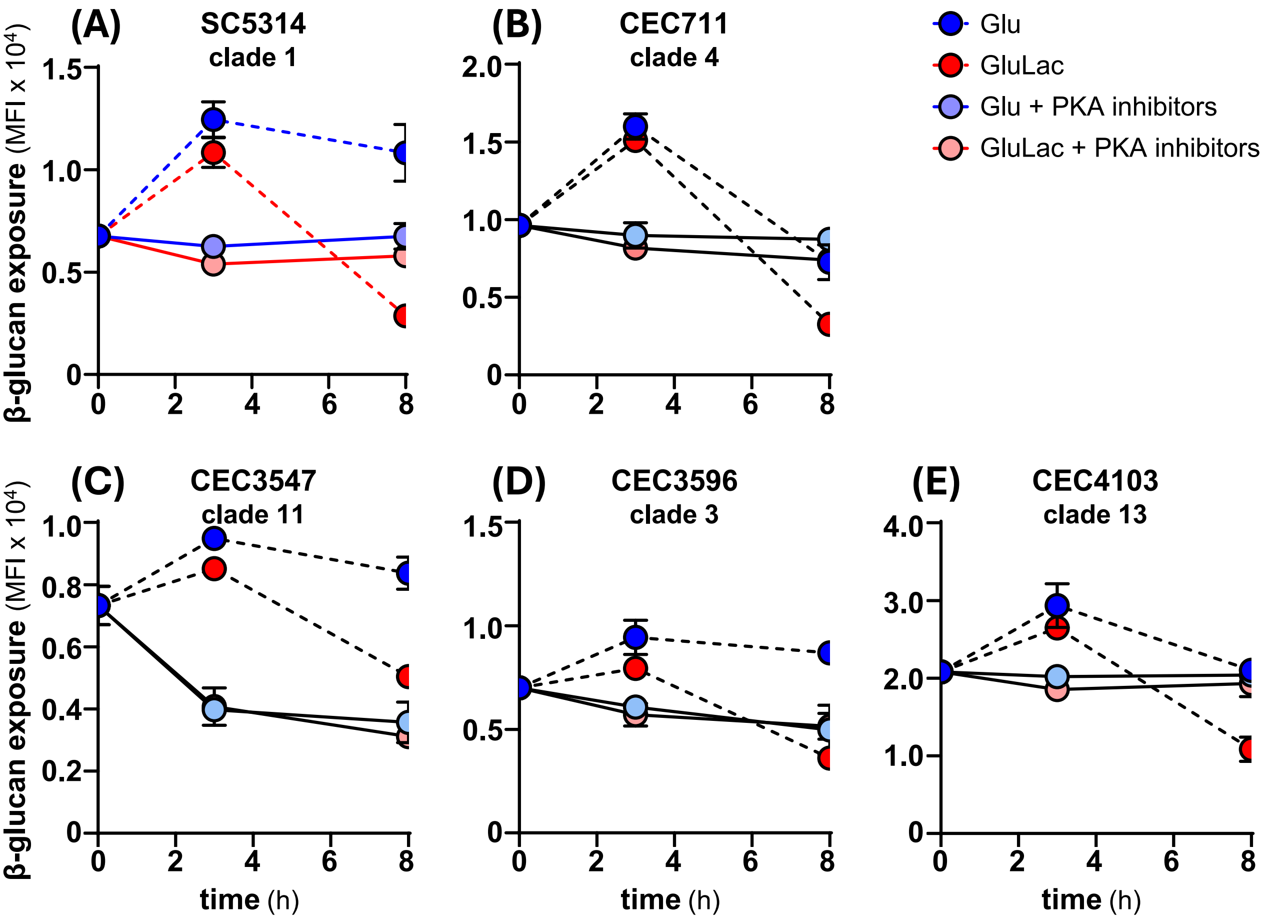

(F)

	Fold lactate-induced masking				
	SC5314	CEC711	CEC3547	CEC3596	CEC4103
NO PKA inhibitors	3.79	2.23	1.66	2.41	1.93
PLUS PKA inhibitors	1.16	1.18	1.15	0.97	1.06

Fig. S3. PKA-dependent induction of lactate-induced β-1,3-glucan masking in divergent clinical isolates of *C. albicans*.
C. albicans isolates were subcultured into SD +/- lactate and +/- inhibitors of PKA signalling: 417 μM MDL12330 plus 25 μM myristoylated PKI were added together at t = 0 [Kjellerup L, *et al.* (2018) *Frontiers Microbiol.* 9: 502]. β-1,3-glucan exposure (MFI) was then quantified at 0, 3 and 8 h. The *C. albicans* isolates tested were: **(A)** SC5314, blood isolate, clade 1; **(B)** CEC711, superficial isolate, clade 4; **(C)** CEC3547, commensal isolate, clade 11; **(D)** CEC3596, invasive isolate, clade 3; **(E)** CEC4103, superficial isolate, clade 13. Each datapoint represents the mean and standard deviation for three independent measurements. **(F)** Fold change in β-1,3-glucan exposure induced by lactate (MFI_{Glu} / MFI_{GluLac} at 8h). The data confirm that: **(i)** the PKA signalling inhibitors mirror the effects of the *tpk1 tpk2* double mutation upon β-1,3-glucan exposure and shaving in the SC5314 background (Fig. 3B); **(ii)** the divergent clinical isolates examined all display lactate-induced β-1,3-glucan shaving; and **(iii)** in all of these isolates, this phenotype is dependent upon PKA signalling.

Supplementary Figure S4

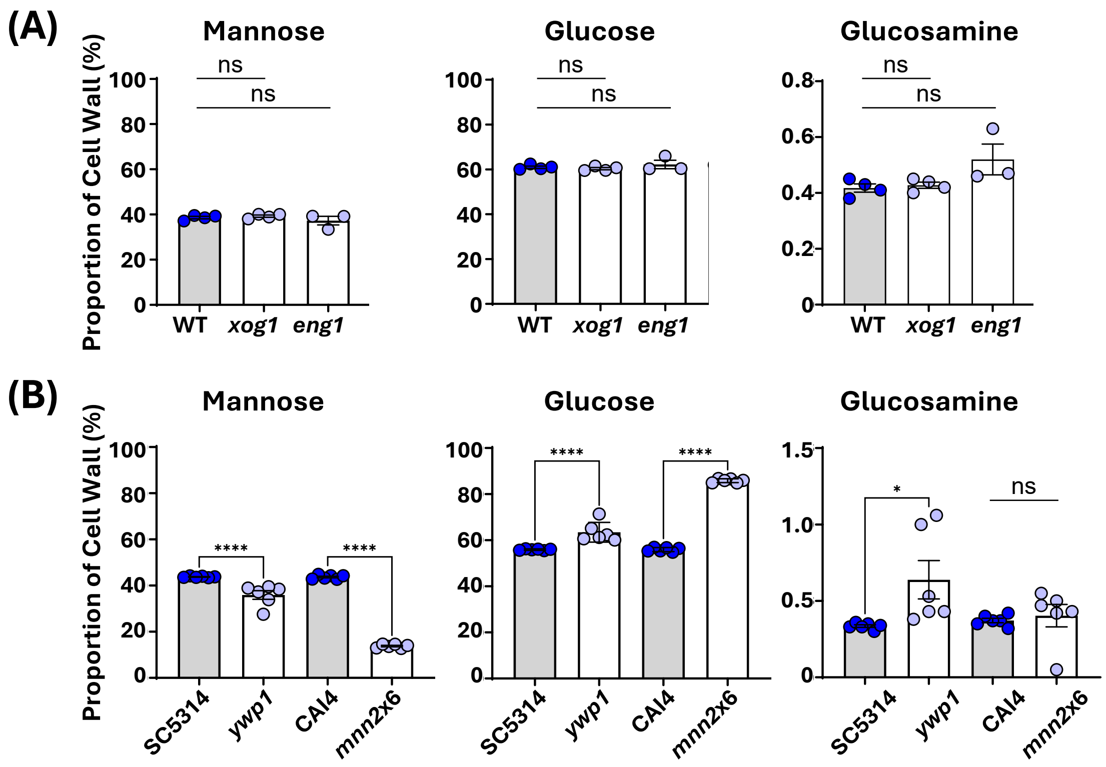

Fig. S4. Impact of mutations on the carbohydrate content of the *C. albicans* cell wall. *C. albicans* mutants and their isogenic wild type controls were grown in SD, harvested in exponential phase, and the carbohydrate content of their cell walls of cells determined by HPIC (Materials and Methods): mannose (mannan); glucose (glucan); glucosamine (chitin). **(A)** Independent *C. albicans xog1* and *eng1* mutants (pale blue) were compared to their independent wild type controls (dark blue) (Table S1). **(B)** *C. albicans ywp1* and *mnn2*x6 mutants (pale blue) were compared to their isogenic wild type controls SC5314 and CAI4, respectively (dark blue) (Table S1). Data represent means and standard deviations from at least three independent measurements. These data were analysed using ordinary one-way ANOVA comparing each mutant to their wild type control: ns, not significant; * $p < 0.05$; ** $p < 0.01$; *** $p < 0.001$; **** $p < 0.0001$.

Supplementary Figure S5

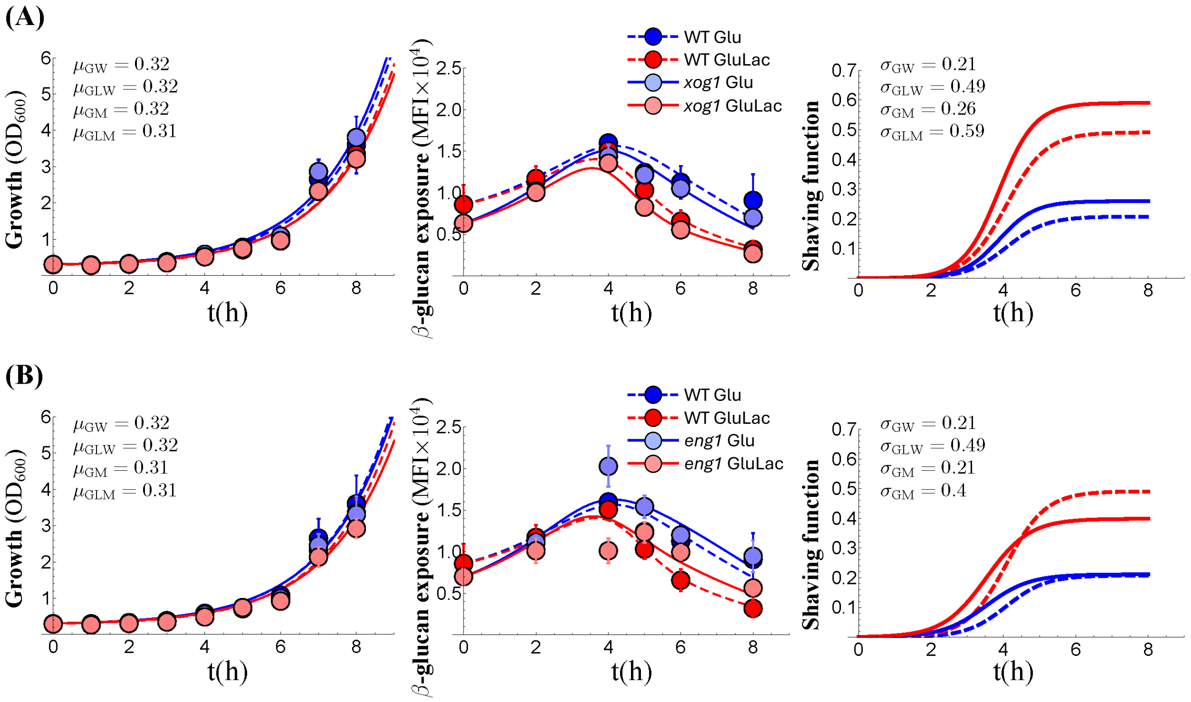

Fig. S5. Influence of Xog1 and Eng1 β-1,3-glucanases on shaving.
Comparison of growth and β-1,3-glucan exposure for *C. albicans xog1* and *eng1* mutants against wild type controls. *Left panels*: growth of wild type controls (dark colours) and mutants (light colours) without lactate (Glu, dark/light blue, μ_{GW}, μ_{GM}) and with lactate (GluLac, red/pink, μ_{GLW}, μ_{GLM}). Means plus standard deviations for four independent mutants and wild type strains are shown with the lines of best fit: wild type, dotted lines; mutants, solid lines. *Middle panels*: dynamics of β-1,3-glucan exposure (MFI) for cells in the same cultures. *Right panels*: shaving functions (σ) for cells in the same cultures: wild type, dotted lines; mutants, solid lines. **(A)** Independent *xog1* strains versus wild type controls: WT A03, WT A08, WT A10, WT A11, *xog1* B07, *xog1* B08, *xog1* B11, *xog1* C10 (Table S1). **(B)** Independent *eng1* strains versus the wild type controls: WT A03, WT A08, WT A10, WT A11, *eng1* C01, *eng1* C08, *eng1* C09, *eng1* D08 (Table S1).

Supplementary Table S1. *C. albicans* strains used in this study

Strain	Name	Parent	Genotype	Source
SC5314	SC5314	-	blood isolate	1
CEC711	711	-	superficial isolate, clade 4	2
CEC3547	3547	-	commensal isolate, clade 11	2
CEC3579	3579	-	commensal isolate, clade 3	2
CEC3596	3596	-	invasive isolate, clade 3	2
CEC3603	3603	-	commensal isolate, clade 1	2
CEC3669	3669	-	superficial isolate, clade 2	2
CEC3683	3683	-	invasive isolate, clade 4	2
CEC4103	4103	-	superficial isolate, clade 13	2
CAI4	CAI4	SC5314	*ura3Δ::imm434/Δura3Δ::imm434*	3
NM23	*gpa1 gpa2*	CAI4	*ura3Δ::imm434/ura3Δ::imm434*, *gpa2Δ::hisG/gpa2Δ::hisG, gpr1Δ::hisG/gpr1Δ::hisG-URA3-hisG*	4
tpk1Δ tpk2Δ	*tpk1 tpk2*	SN152	*arg4Δ/arg4Δ, leu2Δ/leu2Δ, his1Δ/his1Δ, URA3/ura3Δ::imm434, IRO1/iro1Δ::imm434, tpk1::LEU2/ tpk1::FRT, tpk2::HIS1/ tpk2::ARG4*	5
NGY600	*mnn2*x6	CAI4	*ura3Δ::imm434/ura3Δ::imm434*, *mnn2Δ::dpl200/mnn2Δ::dpl200, mnn22Δ::dpl200/mnn2Δ::dpl200, mnn23Δ::dpl200/mnn23Δ::dpl200, mnn24Δ::dpl200/mnn24Δ::dpl200, mnn26Δ::dpl200/mnn26Δ::dpl200, mnn21Δ::dpl200/mnn21Δ::dpl200 RPS1/rps1Δ::*CIp10	6
Ca 2697	*ywp1* A	SC5314	*ywp1Δ::SAT1/ ywp1Δ::SAT1*	this study
Ca 2698	*ywp1* B	SC5314	*ywp1Δ::SAT1/ ywp1Δ::SAT1*	this study
Ca 2699	*ywp1* C	SC5314	*ywp1Δ::SAT1/ ywp1Δ::SAT1*	this study
barcoded strains	below	CAI4	CAI4, *RPS1*-CIp10-Ptet-GTw (*URA3*)	7

**BARCODED *C. ALBICANS* STRAINS**					
***** Genotype:*** CAI4, *RPS1-CIp10-pTET-GW (URA3)*					
**Strain**	**Name**	**Parent**	**Barcode**	**Barcode sequence** (common 5’ sequence-**unique barcode**-common 3’ sequence)	**Source**
Ca 2247	WT A03	CAI4	BC_A03	CGGTGTCGGTCTCGTAG**TACAATAAAGGGCAGGTGCAACCAT**AGAGACCTCGTGGACATC	7
Ca 2249	WT A08	CAI4	BC_A08	CGGTGTCGGTCTCGTAG**CACAGACGACGTAACTTTTATAGGC**AGAGACCTCGTGGACATC	7
Ca 2251	WT A10	CAI4	BC_A10	CGGTGTCGGTCTCGTAG**TGACCAAGCCTGAATAGCGTCATAA**AGAGACCTCGTGGACATC	7
Ca 2253	WT A11	CAI4	BC_A11	CGGTGTCGGTCTCGTAG**CGTGTATTAGAGTAATCGCATCTAG**AGAGACCTCGTGGACATC	7
Ca 2609	*xog1*Δ B07	** *above*	BC_B07	CGGTGTCGGTCTCGTAG**TCCTCTCGGGAGGCCAATAGAAACG**AGAGACCTCGTGGACATC	7
Ca 2611	*xog1*Δ B08	** *above*	BC_B08	CGGTGTCGGTCTCGTAG**TACGCTTGACCCGTGTAGCTGTCAG**AGAGACCTCGTGGACATC	7

Ca 2613	*xog1*Δ B11	** *above*	BC_B11	CGGTGTCGGTCTCGTAG**TGGTTAACACGGAACCTGAGATCCC**AGAGACCTCGTGGACATC	7
Ca 2615	*xog1*Δ C10	** *above*	BC_C10	CGGTGTCGGTCTCGTAG**TAGCACACCTAGCGTGAACCGAGCC**AGAGACCTCGTGGACATC	7
Ca 2617	*eng1*Δ C01	** *above*	BC_C01	CGGTGTCGGTCTCGTAG**TTACCCATTAAGAGCTCAGCGCAAC**AGAGACCTCGTGGACATC	7
Ca 2619	*eng1*Δ C08	** *above*	BC_C08	CGGTGTCGGTCTCGTAG**TCCGTGTTGTACGACGTTAGACAAC**AGAGACCTCGTGGACATC	7
Ca 2621	*eng1*Δ C09	** *above*	BC_C09	CGGTGTCGGTCTCGTAG**TAGTCTACCTCGCATGGCTAAGCGG**AGAGACCTCGTGGACATC	7
Ca 2623	*eng1*Δ D08	** *above*	BC_D08	CGGTGTCGGTCTCGTAG**TATCCAGTCGGCGATAAGTATGCAA**AGAGACCTCGTGGACATC	7
Ca 2704	*xog1*Δ *eng1*Δ B07a	Ca 2609	BC_B07	CGGTGTCGGTCTCGTAG**TCCTCTCGGGAGGCCAATAGAAACG**AGAGACCTCGTGGACATC	This study
Ca 2705	*xog1*Δ *eng1*Δ B07b	Ca 2609	BC_B07	CGGTGTCGGTCTCGTAG**TCCTCTCGGGAGGCCAATAGAAACG**AGAGACCTCGTGGACATC	This study
Ca 2706	*xog1*Δ *eng1*Δ B11	Ca 2613	BC_B11	CGGTGTCGGTCTCGTAG**TGGTTAACACGGAACCTGAGATCCC**AGAGACCTCGTGGACATC	This study
Ca 2707	*xog1*Δ *eng1*Δ C10	Ca 2615	BC_C10	CGGTGTCGGTCTCGTAG**TAGCACACCTAGCGTGAACCGAGCC**AGAGACCTCGTGGACATC	This study

References

1. Gillum AM *et al.* (1984) Isolation of the *Candida albicans* gene for orotidine-5'-phosphate decarboxylase by complementation of *S. cerevisiae ura3* and *E. coli pyrF* mutations. *Molec. Gen. Genet*. 198, 179-182.
2. Avelar GM *et al.* (2024) A CO_2 sensing module modulates β-1,3-glucan exposure in *Candida albicans*. *mBio* 15, e0189823.
3. Fonzi WA & Irwin MY (1993) Isogenic strain construction and gene mapping in Candida albicans. *Genetics* 134, 717-728.
4. Maidan MM *et al.* (2005) Carbon source induced yeast-to-hypha transition in *Candida albicans* is dependent on the presence of amino acids and on the G-protein-coupled receptor Gpr1. *Biochem. Soc. Trans.* 33, 291-293.
5. Cao C *et al.* (2017) Global regulatory roles of the cAMP/PKA pathway revealed by phenotypic, transcriptomic and phosphoproteomic analyses in a null mutant of the PKA catalytic subunit in *Candida albicans*. *Molec Microbiol* 105, 46-64.
6. Hall RA *et al* (2013) The Mnn2 mannosyltransferase family modulates mannoprotein fibril length, immune recognition and virulence of candida albicans. *PLoS Pathog* 9(4): e1003276.
7. Ma Q *et al.* (2024) Impact of secreted glucanases upon the cell surface and fitness of *Candida albicans* during colonisation and infection. *The Cell Surface* 11, 100128.

Table S2. Optimal parameters values (rounded to 2 decimal places) obtained by fitting the model (1) – (4) from the main text to the experimental data on growth and β-1,3-glucan exposure together with corresponding *p*-value.

	μ_{0G}	**p-value**	μ_{maxG}	***p*-value**	μ_{0GL}	***p*-value**	μ_{maxGL}	***p*-value**	η	***p*-value**	σ_G	***p*-value**	σ_{GL}	***p*-value**	**s**	***p*-value**	t_s	***p*-value**
**SC5314<br>Fig 1B**	0.08	3.3E-15	0.6	0	0.07	0	0.58	0	1.67	0	0.11	0	0.35	0	1.23	0	4.51	0
**SC5314<br>Fig 2A**	0.04	0	0.65	0	0.04	0	0.64	0	1.14	0	0.15	1.2E-06	0.51	0	1.31	0	4.03	0
**Ca372<br>Fig 3A**	0.12	0	0.49	0	0.11	4E-15	0.45	0	2.44	0	0.2	0	0.64	0	1.94	0	4.18	0
**gpr1/gpa2<br>Fig 3A**	0.14	5.2E-06	0.43	0	0.11	0.01	0.43	9.2E-12	1.03	0	0.11	1.8E-08	0.15	2.6E-14	0.89	0	1.06	0
**SN152<br>Fig 3B**	0.09	0	0.46	0	0.08	0	0.46	0	1.75	0	0.1	0	0.31	0	1.37	0	4.8	0
**tpk1/tpk2<br>Fig 3B**	0.11	0	0.32	6.7E-16	0.08	0	0.26	0	0.15	9.1E-06	-0.1	1.8E-07	-0.1	7.6E-06	1.12	0	0.77	0
**A03-11<br>Fig 4A**	0.11	0	0.52	0	0.09	0	0.54	0	1.97	0	0.22	0	0.46	0	1.46	0	4.05	0
**xog1/eng1<br>Fig 4A**	0.13	0	0.5	0	0.09	0	0.53	0	2.6	0	0.26	0	0.4	0	2.14	0	4.35	0
**SC5314<br>Fig 4E**	0.08	0	0.58	0	0.06	0	0.58	0	1.19	0	0.12	0	0.47	0	1.14	0	4.41	0
**ywp1<br>Fig 4E**	0.12	0	0.5	0	0.1	0	0.54	0	1.04	0	-0.08	0	0.28	0	1	0	1	0
**Ca372<br>Fig 4F**	0.07	0	0.58	0	0.06	0	0.58	0	1.76	0	0.21	0	0.55	0	1.8	0	3.92	0
**NGY600<br>Fig 4F**	0.12	0	0.48	0	0.13	0	0.44	0	25.06	0	0.05	0	0.21	0	0.77	0	1.15	0

	μ_0	***p*-value**	μ_{max}	***p*-value**	η	***p*-value**	σ	***p*-value**	**s**	***p*-value**	t_s	***p*-value**
**Lactate<br>Fig 1B**	0.1	0	0.1	0	0.67	0	0.04	2.1E-08	0.96	0	4.75	0
**Butyrate<br>Fig 2A**	0.03	0	0.41	0	2.23	0	0.25	0	1.75	0	6.92	0

	μ_{0GN}	***p*-value**	μ_{maxGN}	***p*-value**	μ_{0GH}	***p*-value**	μ_{maxGH}	***p*-value**	η	***p*-value**	σ_{GN}	***p*-value**	σ_{GH}	***p*-value**	**s**	***p*-value**	t_s	***p*-value**
**SC5314<br>Fig 2B**	0.07	0	0.62	0	0.1	0	0.1	0	1.67	1.7E-06	0.3	0.01	0.45	3.4E-08	1.72	9.6E-08	1.98	0
